# Estrogen regulation of microcephaly genes and evolution of brain sexual dimorphism in primates

**DOI:** 10.1186/s12862-015-0398-x

**Published:** 2015-06-30

**Authors:** Lei Shi, Qiang Lin, Bing Su

**Affiliations:** State Key Laboratory of Genetic Resources and Evolution, Kunming Institute of Zoology, Chinese Academy of Sciences, 32 East Jiao-Chang Road, Kunming, 650223 Yunnan PR China; Yunnan Key Laboratory of Primate Biomedical Research, Kunming, 650000 China; Kunming College of Life Science, University of Chinese Academy of Sciences, Beijing, 100101 China

**Keywords:** Sexual dimorphism, Brain size, Brain evolution, Primate, Estrogen

## Abstract

**Background:**

Sexual dimorphism in brain size is common among primates, including humans, apes and some Old World monkeys. In these species, the brain size of males is generally larger than that of females. Curiously, this dimorphism has persisted over the course of primate evolution and human origin, but there is no explanation for the underlying genetic controls that have maintained this disparity in brain size.

**Results:**

In the present study, we tested the effect of the female hormone (estradiol) on seven genes known to be related to brain size in both humans and nonhuman primates, and we identified half estrogen responsive elements (half EREs) in the promoter regions of four genes (MCPH1, ASPM, CDK5RAP2 and WDR62). Likewise, at sequence level, it appears that these half EREs are generally conserved across primates. Later testing via a reporter gene assay and cell-based endogenous expression measurement revealed that estradiol could significantly suppress the expression of the four affected genes involved in brain size. More intriguingly, when the half EREs were deleted from the promoters, the suppression effect disappeared, suggesting that the half EREs mediate the regulation of estradiol on the brain size genes. We next replicated these experiments using promoter sequences from chimpanzees and rhesus macaques, and observed a similar suppressive effect of estradiol on gene expression, suggesting that this mechanism is conserved among primate species that exhibit brain size dimorphism.

**Conclusions:**

Brain size dimorphism among certain primates, including humans, is likely regulated by estrogen through its sex-dependent suppression of brain size genes during development.

**Electronic supplementary material:**

The online version of this article (doi:10.1186/s12862-015-0398-x) contains supplementary material, which is available to authorized users.

## Background

Sexual dimorphism is a common phenomenon in humans and many non-human primate species, such as chimpanzees and rhesus macaques. It is exhibited in a variety of physical characteristics, such as body size, brain size, hair color and skeletal structure, as well as the central nervous system (CNS) [1]. For CNS, the most obvious sexual dimorphism is the size of the brain. Among numerous primates, males have larger brains than females; in humans for example, on average the total brain volume of males is about 11 % larger than in females. Similar differences also exist for regional brain volumes: total grey matter volume (9 % larger), and cerebellum volume (9 % larger) [2–4]. Brain size dimorphism is curiously prominent in apes and humans as well as in some Old World monkey species such as macaques, but no brain size dimorphism is present in New World monkeys (such as common marmoset) or prosimians (such as lorises) [5]. This disparity suggests that the origin of brain size dimorphism in primates is a relatively recent event, one that occurred after the split between Old and New World monkeys about 45 million years ago. Brain size dimorphism also exists in many non-primate mammalian species [6], suggesting multiple origins of this phenomenon during evolution.

One of the hallmarks of primate evolution, and particularly the origin of humans, is an ever-increasing brain size. To date, most studies on brain evolution have focused on understanding the genetic basis of the between-species divergence of brain size among primates, especially between humans and non-human primates [7]. These studies have found a series of genes regulating brain size. In humans, truncated mutations of these genes would cause a rare human genetic disorder, primary microcephaly (MCPH, OMIM#251200), which is characterized by a severe reduction of brain size as well as mild to severe cognitive impairment [8]. The genes purportedly related to MCPH include BRIT1/MCPH1 (BRCT-repeat inhibitor of hTERT expression [9, 10], which encodes microcephalin; locus MCPH1), WDR62 (WD repeat domain 62; MCPH2) [11–13], CDK5RAP2 (cyclin-dependent kinase 5 regulatory associated protein 2; MCPH3) [14], CEP152(centrosomal protein 152 kDa; MCPH4) [15], ASPM(abnormal spindle-like microcephaly-associated protein; MCPH5) [16], CENPJ(centromeric protein J; MCPH6) [14], and STIL(SCL/TAL1 interrupting locus; MCPH7) [17]. Interestingly, all seven MCPH genes encode centrosome or spindle pole proteins taking part in proper centrosome or mitotic microtubule organization [18], indicating their important roles during neurogenesis. Likewise, gene knockout mouse models of three MCPH genes (ASPM, CDK5RAP2 and MCPH1) all resulted in marked reduction in brain size [19–22].

Of the seven identified MCPH genes, four were previously shown to have undergone rapid evolution at the protein sequence level due to Darwinian positive selection during primate evolution, suggesting that these genes contributed to the evolution of the primate brains, and by extension human brains [23, 24]. Among humans, previous studies showed that sequence variations in three of these MCPH genes (ASPM, CDK5RAP2 and MCPH1) are associated with brain size and structure, but in a sex dependent manner [25, 26], implying that MCPH genes may be in some way regulated by sex hormones. If accurate, it stands to reason that the sex-dependent differences in the expression of these genes may contribute to the sexual dimorphism in the brain sizes observed among humans and certain nonhuman primates. Although no reliable data is available to indicate the assumed difference of sex hormone levels in the brain between males and females, it was reported in humans that puberty time (when sex hormones take into effect) plays an important role in shaping up the difference in brain development between males and females [27–29]. Unfortunately, the underlying mechanisms that would explain this effect are still unknown.

Estrogen is known to be a key regulator of development of female reproductive system and secondary sexual characteristics, but it also plays a role in neural functions by engaging in signaling on synaptic proteins, connectivity and synaptic function [30]. It has three forms, including estradiol, estriol and estrone, among which estradiol is the predominant estrogen during reproductive years both in terms of absolute serum levels as well as in terms of estrogenic activity. The estrogen receptor (ER) is a member of the steroid/nuclear receptor super-family of transcription factors that is required for mediating estrogen-induced responses [31]. While a large number of genes can be regulated by estrogen [32], it has never been tested whether MCPH genes are direct targets of estrogen. In this study, we investigated the regulation of estradiol on the seven MCPH genes, and found that four of them (ASPM, CDK5RAP2, MCPH1 and WDR62) can be regulated by estradiol. We then examined the effects of estradiol on the promoter activity of these four MCPH genes and found that estradiol is capable of suppressing the promoter activities of these MCPH genes in both humans and nonhuman primates, and moreover that this suppression mechanism may contribute to the differential development and sexual dimorphic size differences observed between the male and female primate brains.

## Results

### Identification of estrogen responsive elements in four microcephaly genes

**Fig. 1:**
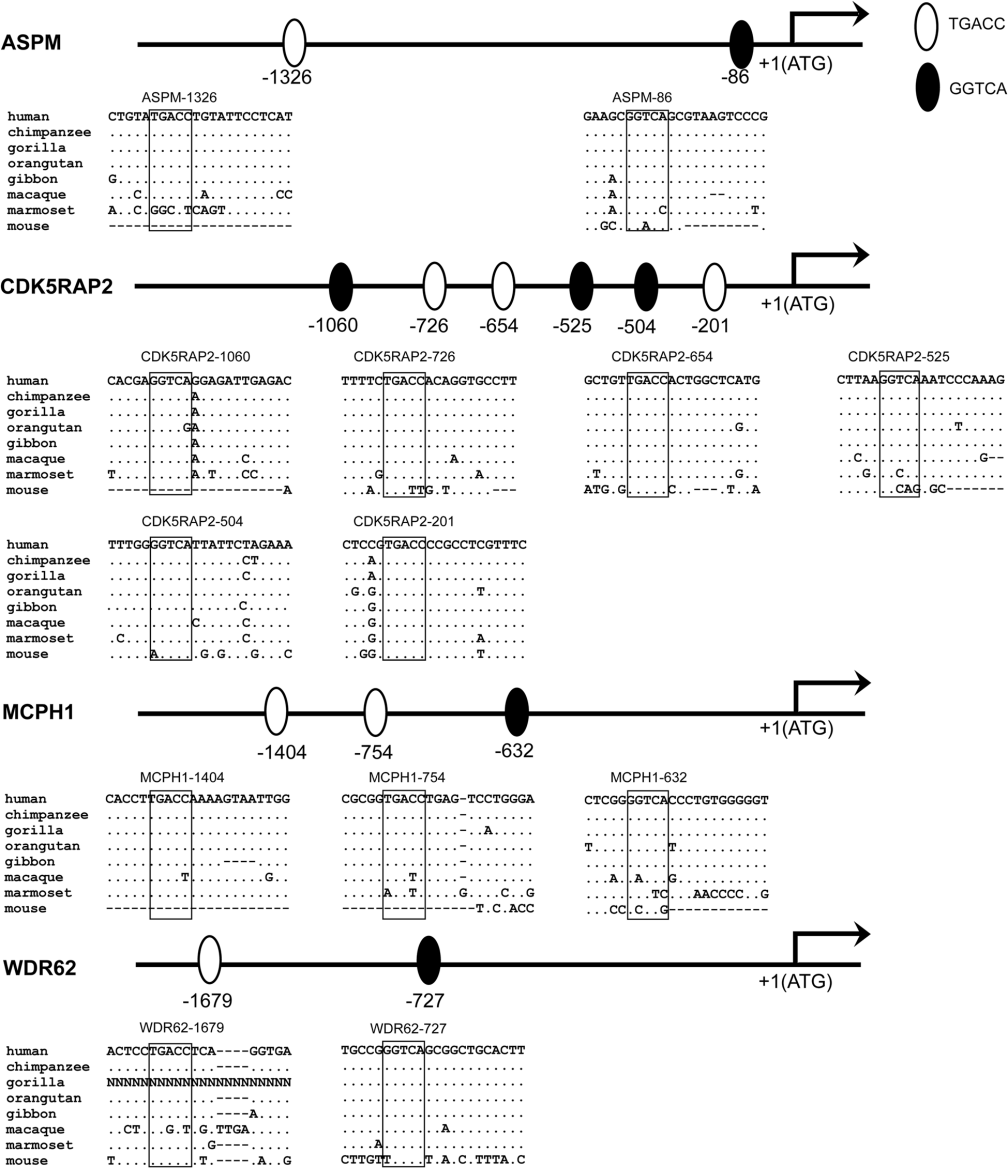
Identification of half EREs in the MCPH gene promoter sequences. The half EREs were mapped based on the gene’s translational start site

To determine whether estradiol could regulate the seven currently known microcephaly genes (ASPM, CENPJ, CEP152, CDK5RAP2, MCPH1, STIL and WDR62), we conducted a search of the potential ERE (estrogen response element) sites in gene promoter regions. Four genes (ASPM, CDK5RAP2, MCPH1 and WDR62) contain predicted half ERE sites (TGACC or GGTCA) in their promoters (Fig. 1) [33, 34]. In total, there are two predicted half ERE sites in ASPM (Fig. 1), six in CDK5RAP2 (Fig. 1), three in MCPH1 (Fig. 1) and two in WDR62 (Fig. 1). Sequence alignments of the promoter regions of representative primate species and mouse indicated that the predicted half ERE sites are, in general, conserved among human and nonhuman primates (Fig. 1), implying functional constraints on these half ERE sites. However, for ASPM and MCPH1, 4 out of the 5 half ERE sites are not conserved in marmosets (Fig. 1).

**Fig. 2:**
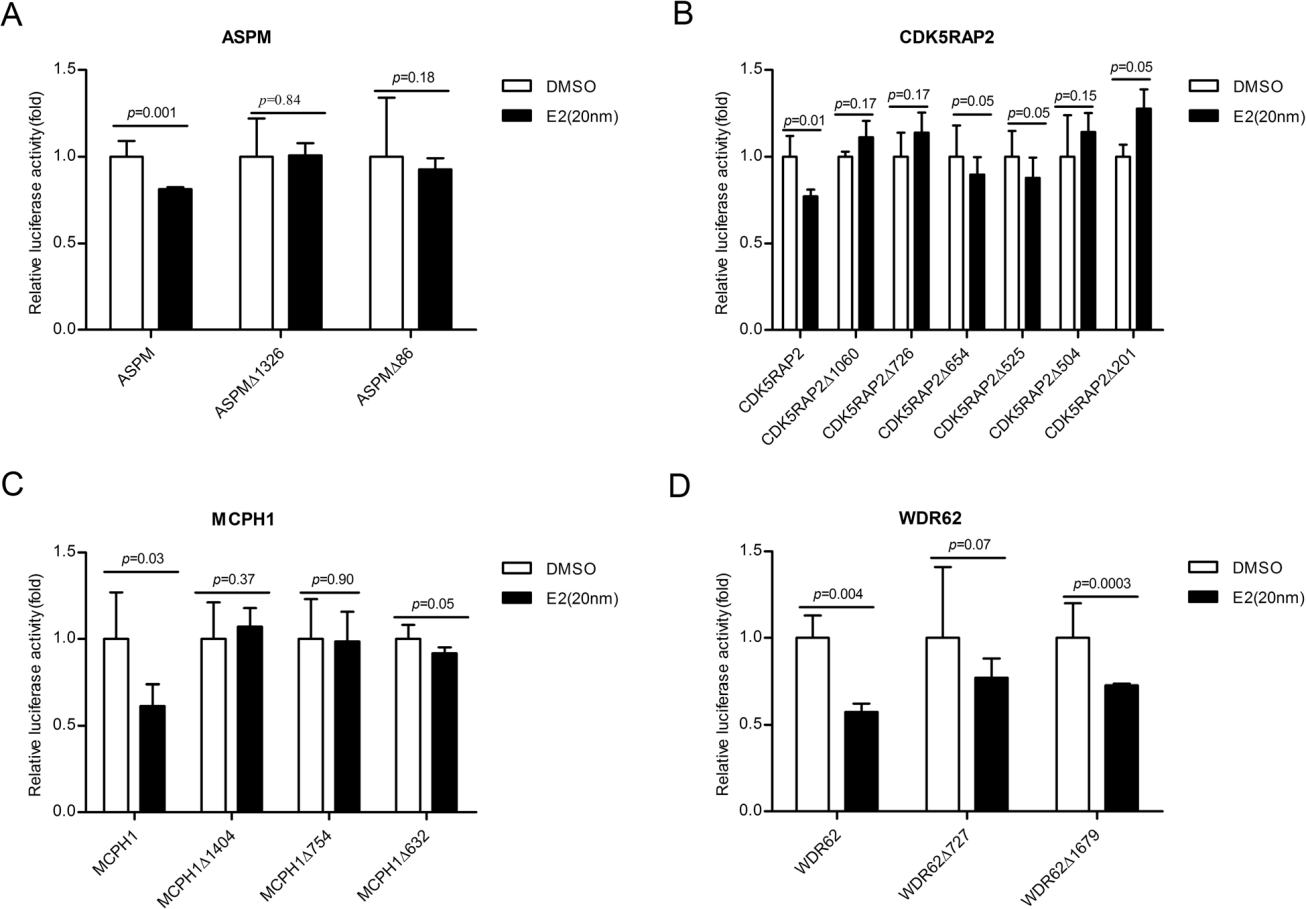
E2 represses the promoter activities of four MCPH genes. **a** Quantification of repressive activity of ASPM gene and the ASPM-1326 and ASPM-86 deletion mutants using luciferase reporter gene assay. **b** Quantification of repressive activity with deletion mutations of half ERE sequence at −1060, −726, −654,-525,-504 and −201 upstream of the CDK5RAP2 translation start site and CDK5RAP2 gene using luciferase reporter gene assay. **c** Quantification of repressive activity with deletion mutations of half ERE sequence at −1404, −754 and −632 upstream of the MCPH1 translation start site and also MCPH1 gene, using luciferase reporter gene assay. **d** Quantification of repressive activity with deletion mutations of half ERE sequence at −727 and −1679 upstream of the WDR62 translation start site and WDR62 gene using luciferase reporter gene assay. The HEK293T cells were transiently transfected with vector containing human ERα

To test whether the predicted half ERE sites are functional, we cloned the promoters of the four genes (Additional file 1: Figure S1; human promoters were used) into luciferase vectors and then assayed their capacity of modulating promoter activity in response to estradiol treatment of the transfected HEK293T cells. The 17-β-estradiol (abbreviated as E2) was used. We found that E2 (20 nm) could regulate the promoter activities of all four microcephaly genes, significantly repressing the promoter activities with the presence of ERα (Fig. 2). On average, the down-regulation levels of the four MCPH genes were 39 % for MCPH1, 43 % for WDR62, 19 % for ASPM and 23 % for CDK5RAP2. We further tested different E2 dosages (1nM-50nM), and the results remained the same (Additional file 1: Figure S1 and Additional file 2: Figure S2).

### Functional validation of half EREs using mutant assays

In order to clarify whether E2’s regulation on the MCPH genes is mediated by direct interaction between E2 and the predicted half ERE sites, we designed a mutant assay by deleting the half ERE sites in the promoters. For ASPM, when either of the two half ERE sites was deleted, the repressive effect disappeared (Fig. 2a), indicating that the two half ERE sites are necessary for ASPM’s response to E2’s regulation. Similarly, for CDK5RAP2, among the six half ERE sites, three showed loss of the repressive effects but the other three did not (Fig. 2. For MCPH1, two of the three half ERE sites showed a loss of repression when deleted (Fig. 2c), while one of the two half ERE sites for WDR62 appeared to be necessary for E2’s regulation (Fig. 2d). Taken collectively, the mutant assay demonstrated that the regulation of E2 on the four MCPH genes is mediated by interaction with the half ERE sites. Interestingly, we did not observe a graded phenotype of the loss of repression in the mutant assays, an implication of a possible cooperative effect among the half ERE sites.

### E2 represses endogenous MCPH gene expression

**Fig. 3:**
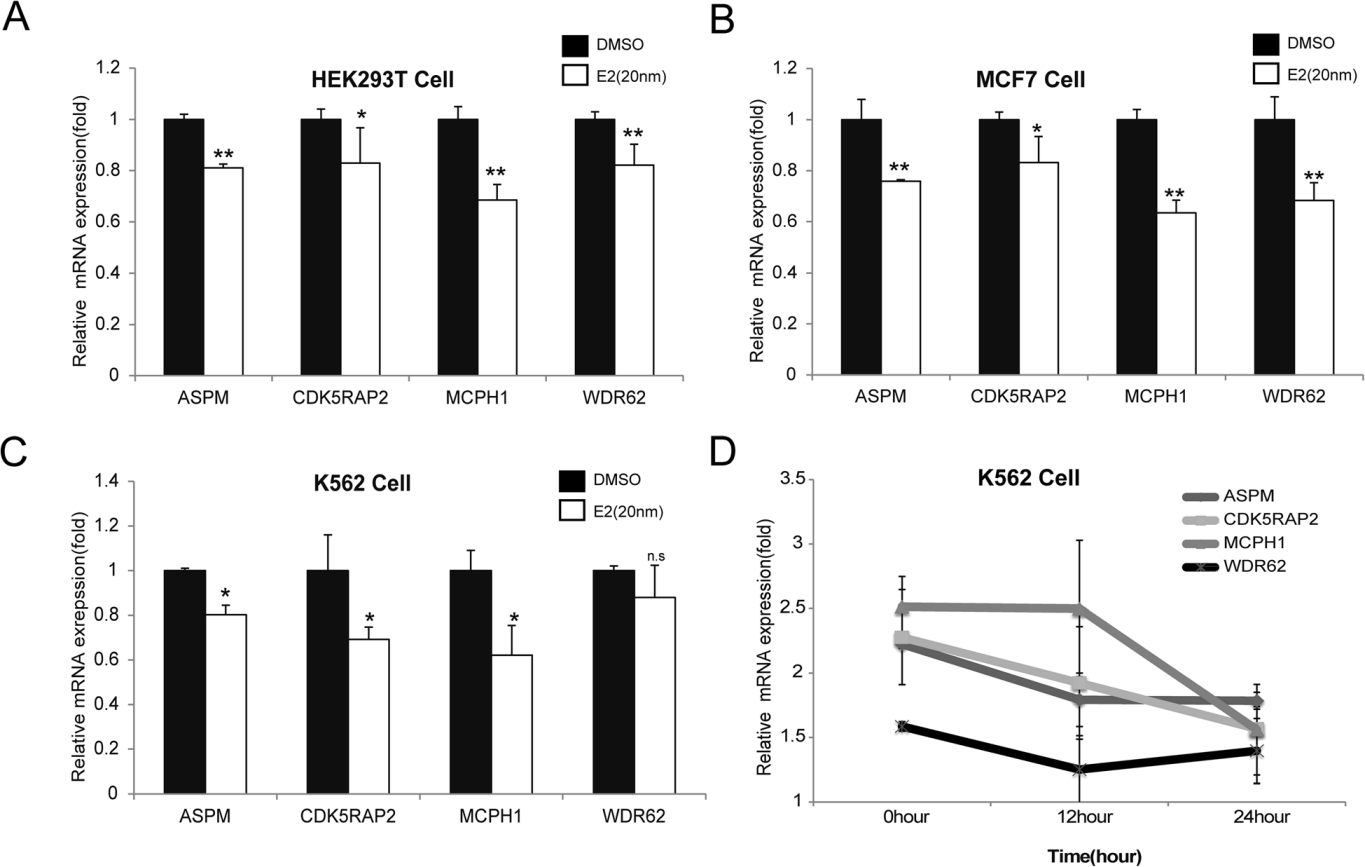
Endogenous MCPH gene expression measurement in human cells treated by E2. **a-c** Quantitative real-time polymerase chain reaction (PCR) measure effects of E2 deprivation (white bars) and E2 (black bars) on endogenous ASPM, CDK5RAP2 MCPH1 gene expression in HEK293, MCF7 and K562 cells, normalized to actin. ASPM, CDK5RAP2, MCPH1 and WDR62 gene expression levels in E2-deprived cells were set as a level of 1-fold expression level. **d** ASPM, CDK5RAP2, MCPH1 and WDR62 mRNA were determined by real-time quantitative PCR in K562 cell lines (ASPM: 12 h, p = 0.06, 24 h, p = 0.002; CDK5RAP2: 12 h, *p* value is 0.34, 24 h, p = 0.06; MCPH1: 12 h, p = 0.96, 24 h, p = 0.02;). The promoter activity was measured as the ratio of luciferase activity, which was normalized by setting the value of the control (DMSO treatment) as 1. All histograms represent the mean ± SD of at least three independent experiments, and each experiment contains three repeats. (*p < 0.05; **p < 0.01; *ns* : not significant)

Given the observed repression of the promoter activities of the four MCPH genes in the reporter gene assays, we tested the effects of E2 on endogenous gene expression. The HEK293T cells capable of expressing estrogen receptors [35] were used to quantify mRNA expression of the four MCPH genes under E2 treatments (DMSO was used as control because E2 is dissolved in DMSO). The results showed that all four MCPH genes were down-regulated endogenously in the HEK293T cells following E2 treatments of 24 h (*p* < 0.05, *t* test) (Fig. 3a). The same effect was also seen in the E2 treated MCF7 cells and K562 cells (*p* < 0.01, *t* test) (Fig. 3b and c). Additionally, time course analysis using K562 cells revealed the same pattern (Fig. 3d), suggesting that E2 can repress the promoter activity of the four MCPH genes.

### Interspecific comparison of E2’s repressing effects in different primate species

To test if the observed E2 regulation of the MCPH genes is conserved across other primate species, we cloned the promoters of the four MCPH genes from chimpanzees and macaques (Additional file 3: Figure S3) and tested their response to E2 treatments. Similar to the results of the human genes, promoters from all four MCPH genes in both chimpanzees and macaques showed repressed activity under E2 treatments (Additional file 4: Figure S4), with the exception of a non-significant repression of the macaque ASPM promoter (Additional file 4: Figure S4E, *p* = 0.07).

**Fig. 4:**
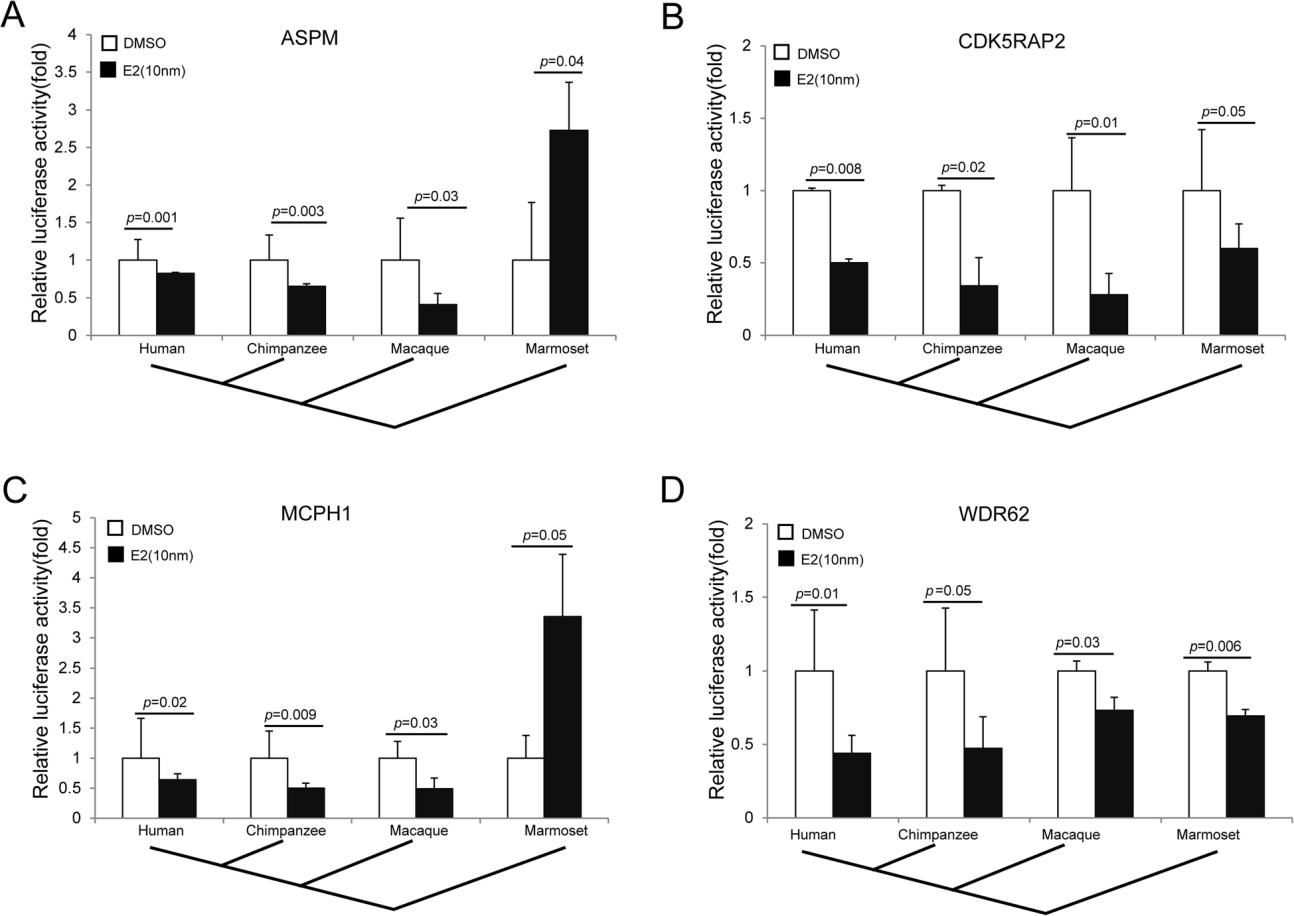
E2 suppresses the promoter activities of MCPH genes in SK-N-SH cells. **a-d** Quantification of promoter activity of ASPM, CDK5RAP2, MCPH1 and WDR62 using luciferase reporter gene assay. The SK-N-SH cells (derived from human neuroblastoma) were transiently transfected with vector containing human ERα

Furthermore, we used a neural cell line (the SK-N-SH cell line derived from human neuroblastoma) and tested all four primate species including marmosets. We observed a similar pattern, in which the repression effects are conserved across humans, chimpanzees and macaques that are known to exhibit sexual dimorphism (Fig. 4). In marmosets, WDR62 and CDK5RAP2 also showed the repression effects (Fig. 4b and d), but ASPM and MCPH1 did not (Fig. 4a and c) because their half ERE sites are not conserved in marmosets (Fig. 1). This observation is consistent with the fact that marmosets do not have brain sexual dimorphism [5].

### Between-sex gene expression comparison in the human brain

To trace the dynamic change of expression of the MCPH genes during brain development, we used currently published human brain transcriptome data (www.brainspan.org) to construct the expression curve of each MCPH gene (Additional file 5: Figure S5). The results showed that there might be potential expression differences between males and females for MCPH1 and CDK5RAP2 (Additional file 5: Figure S5). Further investigation of these genes, particularly MCPH1 and CDK5RAP2 are warranted.

## Discussion

Estrogen is well known to influence numerous sexual dimorphic traits in both humans and several other primates, but its role in brain development has never been clearly articulated. Previous association analysis in humans indicated that three of the brain size regulating genes (ASPM, CDK5RAP2 and MCPH1) contain sequence variations significantly associated with brain size, but more provocatively that the associations are sex-dependent [25, 26], suggesting that sex hormones may be involved in the regulation of MCPH genes, and by extension brain size. Among the seven MCPH genes, four contain half ERE sites in their promoters. Interestingly, these half ERE sites are generally conserved in those primates exhibiting sexual dimorphism, suggesting they play functional roles in these species, including in humans. Both the reporter gene assay and endogenous expression analysis showed that E2 was able to suppress the expression of these four MCPH genes, implying a potentially novel role of estradiol in the observed differences in male and female brain sizes among primates. The mutant assay indicated that the repressive effect of E2 was mediated by its direct interaction with the predicted half ERE sites. Given these results, it is likely that a majority of the currently known brain size regulating genes (4/7) in primates is regulated by estradiol, providing a potential molecular mechanism that explains brain size dimorphism in humans and nonhuman primates.

The four tested MCPH genes are all expressed in the neuroepithelium during fetal development and encode proteins that influence proliferation of neurons in the ventricular zone of the developing brain [8, 18]. Hence, it is likely that the sex difference is established early in development because the size of the neural progenitor pool determines brain size during fetal brain development [36]. A previous report showed that sex-related differences in brain structure are determined mainly by the hormonal environment present during embryonic development [37]. Using published RNAseq data of human fetal brains (http://www.brain-map.org/), we found a large number of differentially expressed genes (2,433 genes) between males and females. In constrast, there are only 412 such genes in the adult brains with little overlap (60 genes) with those in the fetal brains [38] (Additional file 6: Figure S6), supporting the speculated early establishment of sex difference in brain development. Additionally, puberty time also plays an important role in postnatal brain development [27, 28]. It was shown that mean volume of the medial temporal lobe increases in adolescent boys but decreases in adolescent girls with the progression of puberty [29].

Also, it was traditionally argued that females reach puberty earlier than males, therefore, would withdraw earlier from somatic development. Our findings suggest that the female hormone estrogen may serve as one of the key factors causing the between-sex difference of the brain.

A recent study indicated that the sequence evolution of MCPH1 is associated with the evolution of sex dimorphism of anthropoid brain mass [39]. Consistently, in humans, MCPH1 shows a potential between-sex expression divergence at two developmental stages (prenatal and puberty) (Additional file 5: Figure S5) when the levels of estradiol are presumably higher in females than in males. A similar pattern was also seen in CDK5RAP2 during adulthood (Additional file 5: Figure S5). However, these potential differences need further tests. No between-sex difference was detected for the other two MCPH genes (ASPM and WDR62) (Additional file 5: Figure S5). The difference in effect of estradiol suggests that regulation on the MCPH genes by estradiol is likely dependent on spatial-temporal interactions between estradiol and its targets during brain development, though again, more targeted follow-up studies may prove useful in clarifying this possibility. Given the proposed role of MCPH1 in brain size dimorphism, this may explain why New World monkeys like the common marmoset do not exhibit brain size dimorphism [5] because two of the three half ERE sites of MCPH1 are not conserved in the common marmoset (Fig. 1), and no repression effect by estradiol was detected (Fig. 4c).

To date, molecular evolution studies have found that four of the seven known MCPH genes—ASPM, CDK5RAP2, CENPJ and MCPH1—evolved rapidly in primates due to Darwinian positive selection, which is associated with the enlargement of the brain during primate evolution and human origin [23, 24]. For example, the signal of positive selection on MCPH1 was previously inferred in the common ancestor of great apes and humans as well as in the human lineage [24]. Surprisingly, the results of our study here found that three of these fast-evolving MCPH genes overlapped with the four MCPH genes under estradiol regulation, suggesting that natural selection may favor parallel changes of both brain size change and brain size dimorphism. This fits quite well with what we already know, that estradiol is essential for brain development and it can promote neurite growth and regulate synaptic patterning [40]. Ultimately then, when natural selection pushes the enlargement of the brain, it may be that it tends to act on genes already under estradiol regulation, and our results suggest that it does so potentially by suppressing the expression of brain size related genes in females [40].

It should be noted that besides the MCPH genes tested in this study, there might be other MCPH genes or unknown regulatory factors contributing to brain sexual dimorphism in primates. For example, the male sex hormone (testosterone) may play a role in brain sexual dimorphism. Also, why natural selection would favor a relatively smaller brain size in females is not clear. One possible explanation is the differential social roles of males and females that would lead to differential selective pressures during evolution. More evidence needs to be collected to test this hypothesis.

## Conclusions

We demonstrated that estrogen could down-regulate four of the known brain size regulating genes. This suppression effect may contribute to between-sex difference in brain size in humans and nonhuman primates with brain sexual dimorphism, suggesting that sex hormones may play an important role in primate brain evolution.

## Methods

### Cell culture

The HEK293,MCF-7, K562, HL60 and SK-N-SH cell lines were obtained from ATCC. These cell lines were cultured in phenol red-free DMEM (Gibco, Rockville, MD) with 10 % fetal bovine serum (Hyclone, Logan, UT) at 37 °C in a humidified atmosphere containing 5 % CO_2_. For estrogen assays, before treatments, the cells were maintained in phenol red-free DMEM containing 10 % dextran-coated charcoal-stripped fetal bovine serum (DCC-FBS) (Hyclone) for a minimum of 3 days with the media changed every day. Then the cells were treated with E2 (20nM, dissolved in DMSO) or DMSO as control for 24 h.

### Cloning of the human, chimpanzee and macaque MCPH gene promoters

The promoters of MCPH1 were cloned in our previous study [41], or ASPM, MCPH1, CDK5RAP2 and WDR62, promoters from human, chimpanzee, macaque and marmoset were amplified from genomic DNA, and then the >2 kb amplicons were cloned into the pGL3 basic firefly luciferase vector (Promega). Primers used for cloning of ASPM, MCPH1, CDK5RAP2, WDR62 gene 5′-flanking region are shown in Additional file 7: Table S1. XhoI/HinDIII and KpnI/NheI restriction sites were respectively introduced in the forward and reverse primer, and employed for cloning the deletion fragments upstream of the luciferase reporter gene plasmid. The amplified DNA fragment was digested with XhoI/HinDIII and KpnI/NheI (Fermentas, Hanover, MD) and cloned into the pGL3-basic firefly luciferase reporter vector (Promega, Madison, WI) to construct human-aspm, chimpanzee-aspm, macaque-aspm, marmoset-aspm, human-cdk5rap2, chimapanzee-cdk5rap2, macaque-cdk5rap2, macrmoset-cdk5rap2, marmoset-MCPH1, human-wdr62, chimpanzee-wdr62, macaque-wdr62, marmoset-wdr62. The sequences of the cloned DNAs were verified by sequencing the entire region.

### MCPH gene promoter sequence alignment

The microcephaly gene promoter sequences of human, chimpanzee and macaque were obtained from the NCBI (http://www.ncbi.nlm.nih.gov), EMBL (http://www.ensembl.org). Orthologous sequences were aligned using Muscle in MEGA5 (http://www.megasoftware.net) and Clustal W 7.0.5.2 (BioEdit).

### Mutant human MCPH gene promoter constructs

The cloning of promoter constructs in luciferase reporter vector, the pGL3 basic vector and the ASPM, CDK5RAP2, MCPH1 and WDR62 promoter constructs containing mutations in estrogen receptor binding sites were conducted using QuickChange II XL site-directed mutagenesis kit (Stratagene. La Jolla, CA, USA) following the recommended protocols. The sequences of the mutant cloned DNAs were verified by sequencing. Primers used for generating ASPM, CDK5RAP2, MCPH1, WDR62 promoter mutants are listed in Additional file 8: Table S2.

### Transient transfection and luciferase reporter assay

To determine whether E2 regulates the microcephaly genes’ promoter activity, all transfections were carried out in triplicate in 24-well plates (Corning, NY, USA). About 2 × 10^5^ cells were seeded for 24 h prior to transfection. Equal numbers of cells were plated in 24-well and 6-well plates and grown to 80 % confluence. HEK293T, MCF7 and SK-N-SH cells were transfected with the indicated amounts of microcephaly promoter constructs, ERα receptor expression plasmid (kindly provided by Leigh C. Murphy, University of Manitoba) including pTK-Renilla as an internal control, using Lipofectamine 2000 (Invitrogen). After 6 h, the cells were treated with E2 and DMSO for 24 h.

Luciferase activity was assayed 24 h after transfection. The luciferase activity in cell extracts was determined by Dual-luciferase Reporter Assay System (Promega, Madison, WI) according to the manufacturer’s protocols. The relative light units were measured using a luminometer. Each experiment was repeated at least three times to ensure accuracy. The promoter activity was measured as the ratio of luciferase activity, which was normalized by setting the value of the control (DMSO treatment) as 1. All histograms represent the mean ± SD of at least three independent experiments, and each experiment contains six repeats (*p < 0.05; **p < 0.01; *ns* : not significant).

### RT-PCR

Total RNA was extracted using TRIzol (Invitrogen, Carlsbad,CA). The RNA was treated with DNase I (Fermentas) to remove possible genomic DNA contamination, then subjected to reverse transcription using an oligo-dT(20) primer and Ominicript Reverse transcriptase (Qiagen, Valencia, CA).

### MCF-7 real time quantitative PCR

To determine the effect of estrogen on the endogenous MCPH gene expression, MCF-7 cells were treated with E2 (20nM) and control DMSO (same amount volume) for 24 h. These RNAs were reverse transcribed with an oligo-dT(20) primer and amplified with real-time PCR primers. Real time PCR reactions (15-ul total volume containing 0.5-ul 10um primer,7.5-ul SYBR Green Dye (Bio-Rad, CA, USA), and 2-ul of cDNAs) were carried out with a DNA Engine Opticon Continuous Fluorescence Detection System(MJ Research, Waltham MA) for ~40 cycles. Ct values for each gene amplification were normalized by subtracting the Ct value calculated for actin. The normalized gene expression values were expressed as the relative quantity of ASPM, CDK5RAP2, MCPH1, WDR62 gene-specific messenger RNA (mRNA). The oligonucleotide primers used in the real-time quantitative PCR amplifications are shown in Additional file 9: Table S3.

### K562 time series real time quantitative PCR

K562 cells were treated with E2(20nM) and control DMSO(the same amount volume) for 0, 24, 48 h. These RNAs were reverse transcribed with oligo-dT(20) primer and amplified by real-time PCR primers. Real-time PCR reactions (15-ul total volume containing 0.5-ul 10um primer,7.5d-ul SYBR Green Dye from Bio-Rad (CA, USA), and 2-ul of cDNAs) were carried out with a DNA Engine Opticon Continuous Fluorescence Detection System (MJ Research, Waltham. MA) for ~40 cycles. Ct values for each gene amplification were normalized by subtracting the Ct value calculated for GAPDH. The normalized gene expression values were expressed as the relative quantity of ASPM, CDK5RAP2, MCPH1, WDR62 gene-specific messenger RNA (mRNA).

### Human brain development expression data analysis

We downloaded human brain development expression RNAseq data of ASPM, CDK5RAP2, MCPH1 and WDR62 gene from the Atlas of the Developing Human Brain (www.brainspan.org) covering the developing stages ranging from 5–7 post-conceptional weeks (pcw) to over 40 years of age (Additional file 10: Table S4). The RPKM (reads per kilobase per million) value was used to indicate the expression level of ASPM, CDK5RAP2, MCPH1 and WDR62 gene. We divided the brain developmental stages into prenatal time and postnatal. However, due to the sampling strategy of the database, the data is not exactly point to point between males and females.

### Prenatal brain gene expression analysis

We downloaded RNAseq data of human fetal brains from the Atlas of the Developing Human Brain (www.brainspan.org) covering 8–26 post-conception weeks (pcw). Differentially expressed genes between males and females were determined with the DEseq package [42], by modeling count data using a negative binomial distribution. First, size factors are calculated by taking into account the total number of reads in different samples. Second, a dispersion parameter is determined for each gene accounting for biological variation between samples. Finally, a negative binomial distribution is used to fit the counts for each gene. The *p*-value is calculated based on the fold changes. The *p* values adjusted for multiple testing was calculated using the Benjamini-Hochberg procedure, which controls false discovery rate (FDR).

### Statistical analysis

Statistical analyses were performed using Prism 5 (GraphPad); Data was analyzed using the two-tailed Student’s *t* test. *P*-values <0.05 were considered as statistically significant. ANOVA analysis was performed using the R program (http://www.r-project.org/).

## Additional files

Additional file 1: Figure S1. Different dosages of E2 (1nM-50nM) repress the promoter activity of MCPH1. The promoter activity was measured as the ratio of luciferase activity by setting the value of the internal control (empty vector) as one. All histograms represent the mean ± SD of at least three independent experiments, and each experiment includes six repeats. (**p* < 0.05; ***p* < 0.01; ns : not significant). Additional file 2: Figure S2. The repression effect of E2 (5nM) on the promoter activities of the four MCPH genes. HEK293T cells were transiently transfected with empty vector and vector containing human ERα. Cells were treated with 5nM E2 or the same volume DMSO for 36 hours prior to assaying reporter activity using dual-luciferase assays. All histograms represent the mean ± SD of at least three independent experiments, and each experiment include six repeats. (**p* < 0.05; ***p* < 0.01; ns : not significant). Additional file 3: Figure S3. Promoter activity tests of the MCPH genes of chimpanzee and rhesus macaque using reporter gene assays. The promoter activity was measured as the ratio of luciferase activity, which was normalized by setting the value of the internal control (empty vector) as one. Additional file 4: Figure S4. E2 represses the promoter activities of chimpanzee and macaque MCPH genes. (A-D) Quantification of repressive activity of chimpanzee ASPM, CDK5RAP2, MCPH1 and WDR62 promoter using luciferase reporter gene assay. (E-H) Quantification of repressive activity of macaque ASPM, CDK5RAP2, MCPH1 and WDR62 promoter using luciferase reporter gene assay. HEK293T cells were transiently transfected with vector containing human ERα. Cells were treated with 20nM E2 or the same volume DMSO for 36 h prior to assaying reporter activity using dual-luciferase assays. All histograms represent the mean ± SD of at least three independent experiments, and each experiment contains six repeats. (*p < 0.05; **p < 0.01; ns : not significant). Additional file 5: Figure S5. Expression changes of the MCPH genes during human brain development. (A-D) Curve of ASPM, CDK5RAP2, MCPH1 and WDR62 expression changes in PFC during human brain development. The shaded areas indicate the developmental stages showing up-regulation of CDK5RAP2 and MCPH1 in males compared with females. Additional file 6: Figure S6. Comparison of the between-sex differentially expressed genes in the prenatal and postnatal (adult) human brains. Additional file 7: Table S1. Primers used for cloning the 5′-flanking region of the microcephaly genes. Additional file 8: Table S2. Primers used for generating estrogen receptor binding site mutants of the MCPH gene promoter constructs. Additional file 9: Table S3. Primers used for real-time quantitative PCR. Additional file 10: Table S4. The brain samples of different developmental stages used in this study from published data at www.brainspan.org.
