# Supplementary material for: Estrogen regulation of microcephaly genes and evolution of brain sexual dimorphism in primates
Source: BMC Evol Biol. 2015 Jun 30;15:127. doi: 10.1186/s12862-015-0398-x (PMC4487212; doi:10.1186/s12862-015-0398-x)

**Figure. S1.** **Different dosages of E2 (1nM-50nM) repress the promoter activity of MCPH1.** The promoter activity was measured as the ratio of luciferase activity by setting the value of the internal control (empty vector) as one. All histograms represent the mean ± SD of at least three independent experiments, and each experiment includes six repeats. (**p*<0.05; ***p*<0.01; ns : not significant).


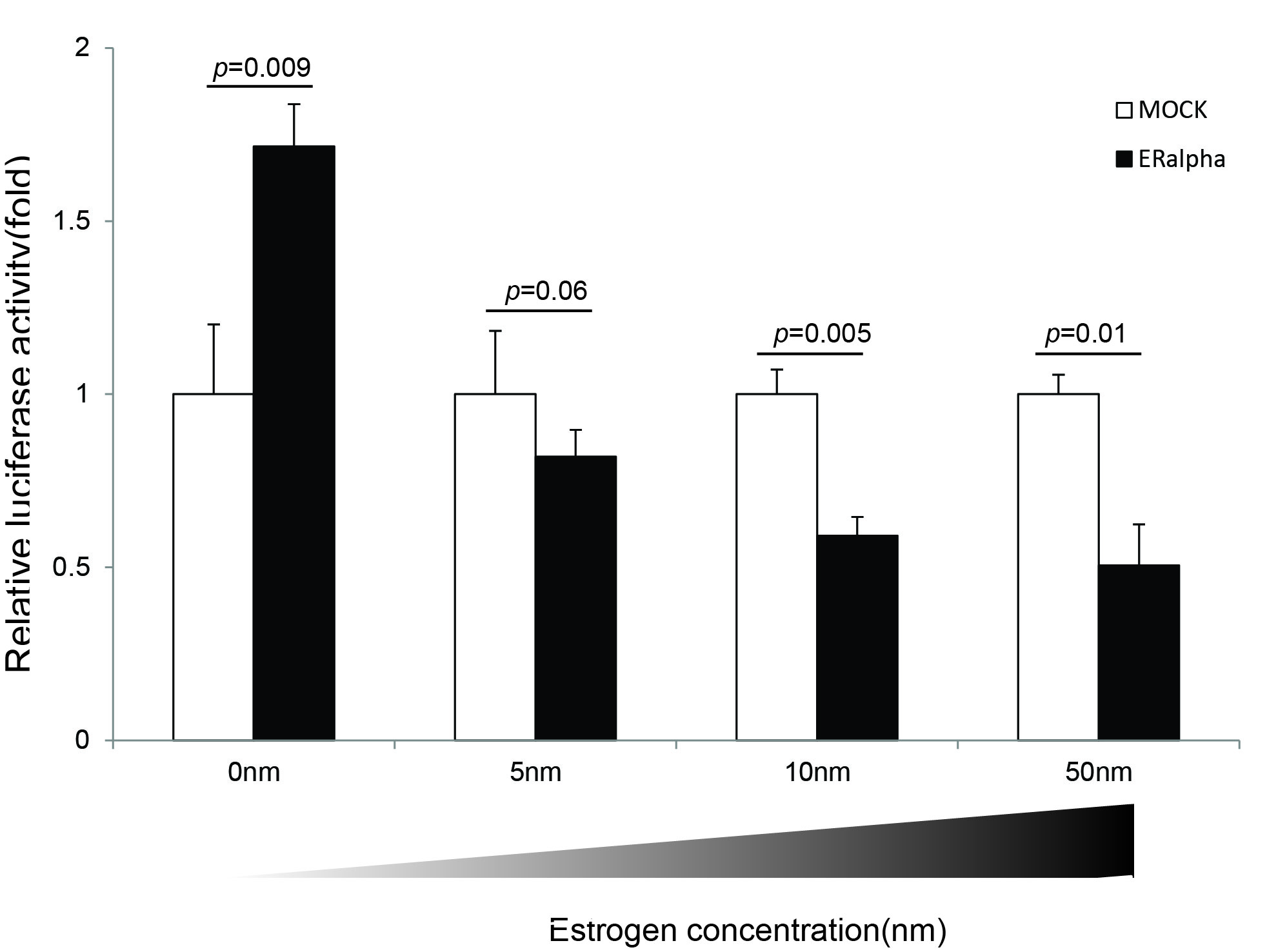

Supplement: Additional file 1: Figure S1. — Different dosages of E2 (1nM-50nM) repress the promoter activity of MCPH1. The promoter activity was measured as the ratio of luciferase activity by setting the value of the internal control (empty vector) as one. All histograms represent the mean ± SD of at least three independent experiments, and each experiment includes six repeats. (*p < 0.05; **p < 0.01; ns : not significant). [file 12862_2015_398_MOESM1_ESM.docx]
