# Supplementary material for: Estrogen regulation of microcephaly genes and evolution of brain sexual dimorphism in primates
Source: BMC Evol Biol. 2015 Jun 30;15:127. doi: 10.1186/s12862-015-0398-x (PMC4487212; doi:10.1186/s12862-015-0398-x)

**Figure. S2.** **The repression effect of E2 (5nM) on the promoter activities of the four MCPH genes.** HEK293T cells were transiently transfected with empty vector and vector containing human ERα. Cells were treated with 5nM E2 or the same volume DMSO for 36 hours prior to assaying reporter activity using dual-luciferase assays. All histograms represent the mean ± SD of at least three independent experiments, and each experiment include six repeats. (**p*<0.05; ***p*<0.01; ns : not significant).


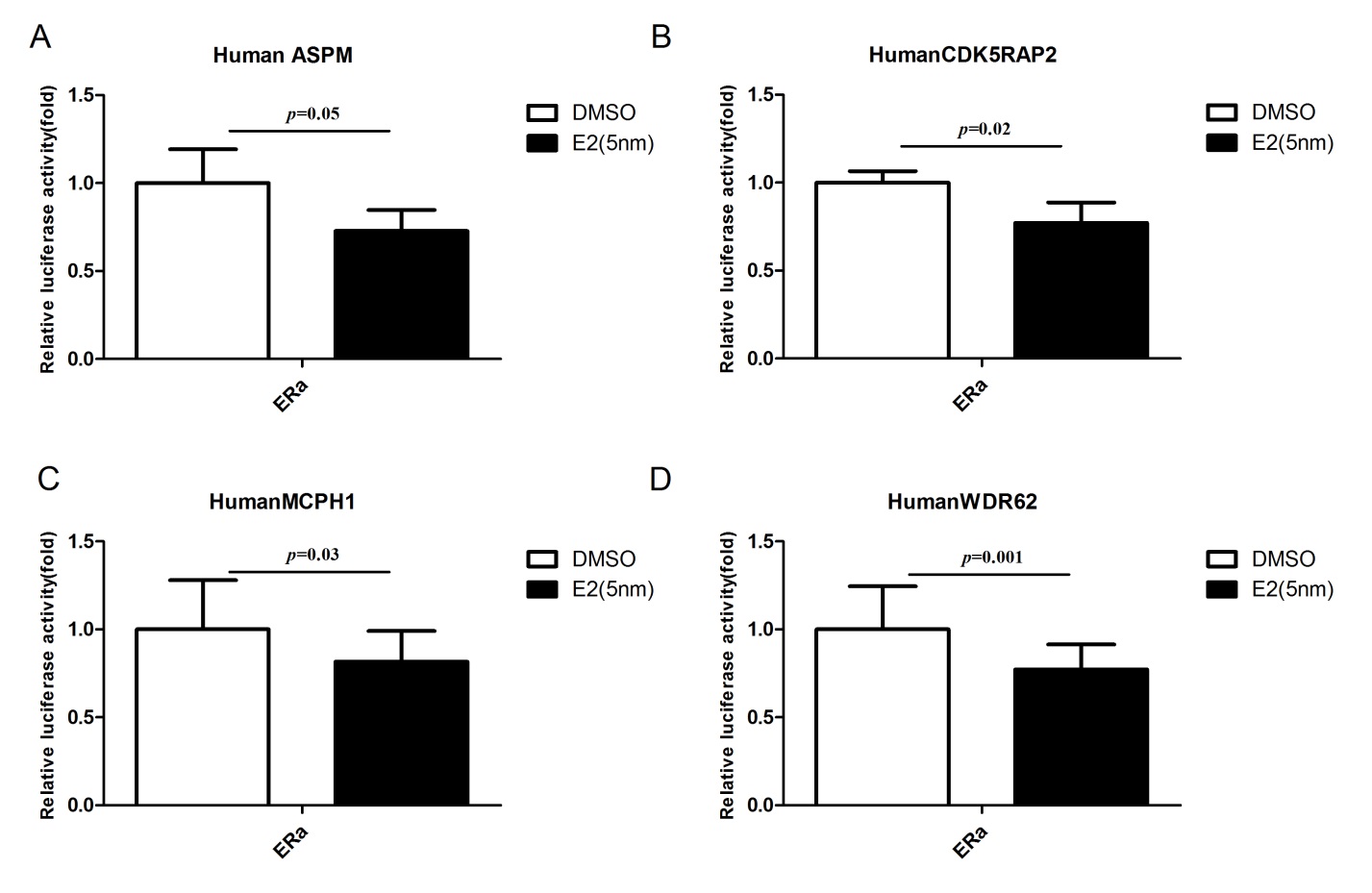

Supplement: Additional file 2: Figure S2. — The repression effect of E2 (5nM) on the promoter activities of the four MCPH genes. HEK293T cells were transiently transfected with empty vector and vector containing human ERα. Cells were treated with 5nM E2 or the same volume DMSO for 36 hours prior to assaying reporter activity using dual-luciferase assays. All histograms represent the mean ± SD of at least three independent experiments, and each experiment include six repeats. (*p < 0.05; **p < 0.01; ns : not significant). [file 12862_2015_398_MOESM2_ESM.docx]
