# Supplementary material for: Estrogen regulation of microcephaly genes and evolution of brain sexual dimorphism in primates
Source: BMC Evol Biol. 2015 Jun 30;15:127. doi: 10.1186/s12862-015-0398-x (PMC4487212; doi:10.1186/s12862-015-0398-x)
Supplement: Additional file 3: Figure S3. — Promoter activity tests of the MCPH genes of chimpanzee and rhesus macaque using reporter gene assays. The promoter activity was measured as the ratio of luciferase activity, which was normalized by setting the value of the internal control (empty vector) as one. [file 12862_2015_398_MOESM3_ESM.docx]

**Figure. S3.** **Promoter activity tests of the MCPH genes of chimpanzee and rhesus macaque using reporter gene assays.** The promoter activity was measured as the ratio of luciferase activity, which was normalized by setting the value of the internal control (empty vector) as one.

**

**
