# Supplementary material for: Estrogen regulation of microcephaly genes and evolution of brain sexual dimorphism in primates
Source: BMC Evol Biol. 2015 Jun 30;15:127. doi: 10.1186/s12862-015-0398-x (PMC4487212; doi:10.1186/s12862-015-0398-x)
Supplement: Additional file 4: Figure S4. — E2 represses the promoter activities of chimpanzee and macaque MCPH genes. (A-D) Quantification of repressive activity of chimpanzee ASPM, CDK5RAP2, MCPH1 and WDR62 promoter using luciferase reporter gene assay. (E-H) Quantification of repressive activity of macaque ASPM, CDK5RAP2, MCPH1 and WDR62 promoter using luciferase reporter gene assay. HEK293T cells were transiently transfected with vector containing human ERα. Cells were treated with 20nM E2 or the same volume DMSO for 36 h prior to assaying reporter activity using dual-luciferase assays. All histograms represent the mean ± SD of at least three independent experiments, and each experiment contains six repeats. (*p < 0.05; **p < 0.01; ns : not significant). [file 12862_2015_398_MOESM4_ESM.docx]

**Figure. S4. E2 represses the promoter activities of chimpanzee and macaque MCPH genes.**

(A-D) Quantification of repressive activity of chimpanzee ASPM, CDK5RAP2, MCPH1 and WDR62 promoter using luciferase reporter gene assay. (E-H) Quantification of repressive activity of macaque ASPM, CDK5RAP2, MCPH1 and WDR62 promoter using luciferase reporter gene assay. HEK293T cells were transiently transfected with vector containing human ERα. Cells were treated with 20nM E2 or the same volume DMSO for 36 h prior to assaying reporter activity using dual-luciferase assays. All histograms represent the mean ± SD of at least three independent experiments, and each experiment contains six repeats. (*p<0.05; **p<0.01; ns : not significant).
