# Supplementary material for: Estrogen regulation of microcephaly genes and evolution of brain sexual dimorphism in primates
Source: BMC Evol Biol. 2015 Jun 30;15:127. doi: 10.1186/s12862-015-0398-x (PMC4487212; doi:10.1186/s12862-015-0398-x)

**Figure S5. Expression changes of the MCPH genes during human brain development.** (A-D) Curve of ASPM, CDK5RAP2, MCPH1 and WDR62 expression changes in PFC during human brain development. The shaded areas indicate the developmental stages showing up-regulation of CDK5RAP2 and MCPH1 in males compared with females


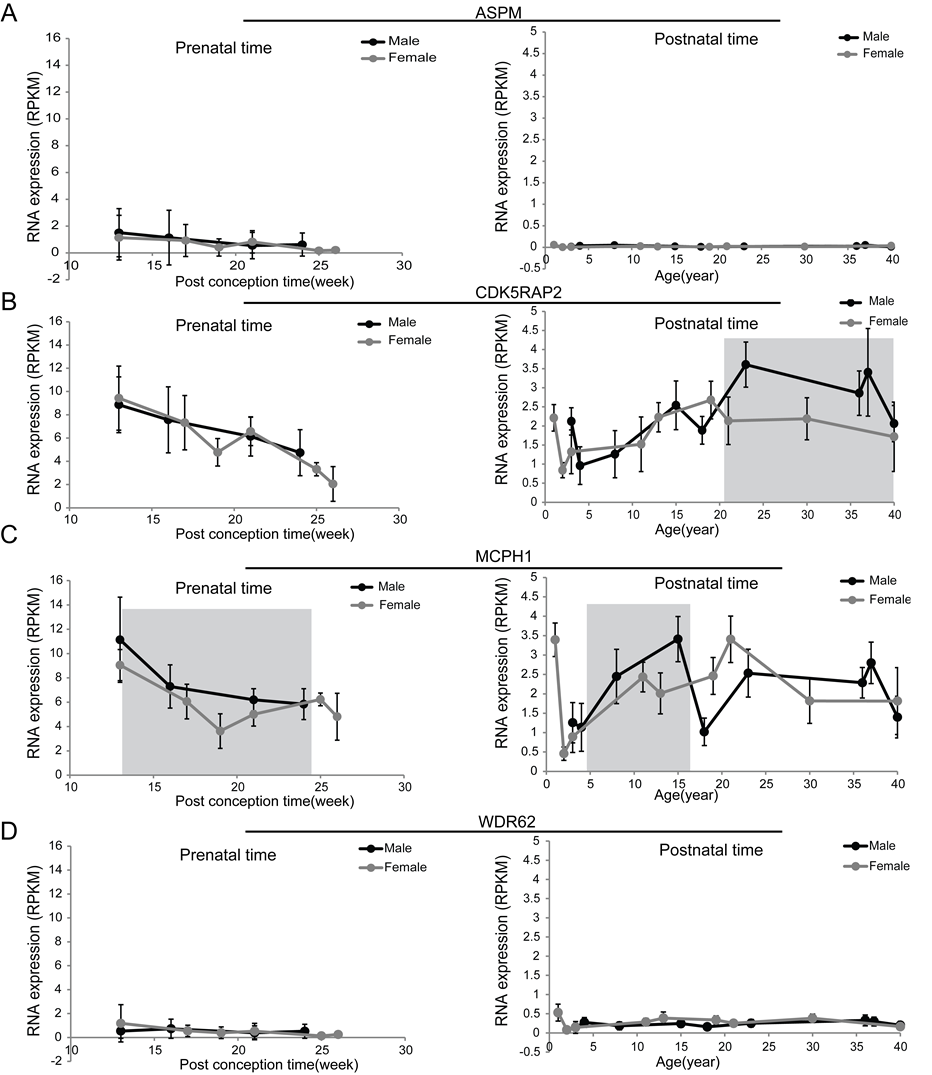

Supplement: Additional file 5: Figure S5. — Expression changes of the MCPH genes during human brain development. (A-D) Curve of ASPM, CDK5RAP2, MCPH1 and WDR62 expression changes in PFC during human brain development. The shaded areas indicate the developmental stages showing up-regulation of CDK5RAP2 and MCPH1 in males compared with females. [file 12862_2015_398_MOESM5_ESM.docx]
