# Supplementary material for: Estrogen regulation of microcephaly genes and evolution of brain sexual dimorphism in primates
Source: BMC Evol Biol. 2015 Jun 30;15:127. doi: 10.1186/s12862-015-0398-x (PMC4487212; doi:10.1186/s12862-015-0398-x)
Supplement: Additional file 6: Figure S6. — Comparison of the between-sex differentially expressed genes in the prenatal and postnatal (adult) human brains. [file 12862_2015_398_MOESM6_ESM.docx]

**Figure. S6. Comparison of the between-sex differentially expressed genes in the prenatal and postnatal (adult) human brains.**

**
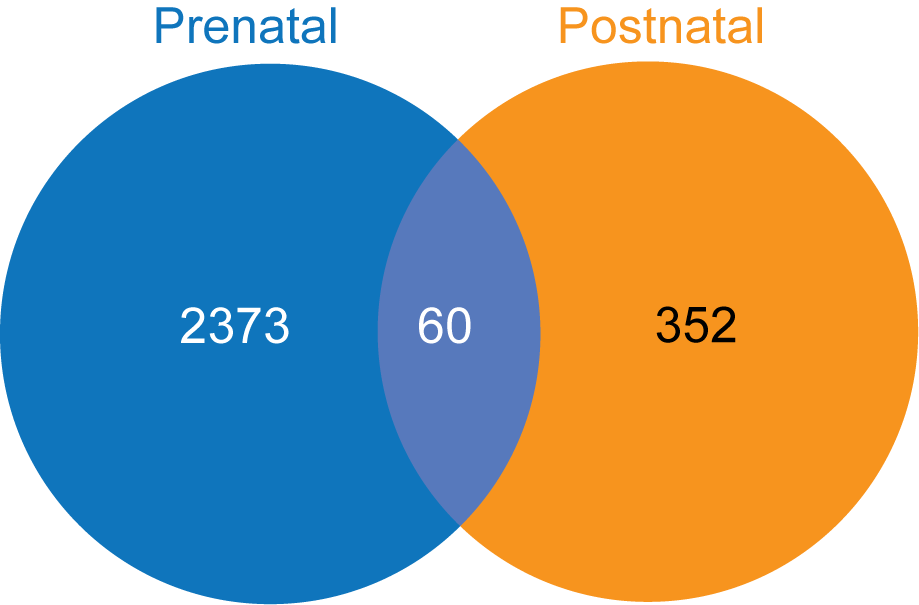
**
