# Supplementary material for: Estrogen regulation of microcephaly genes and evolution of brain sexual dimorphism in primates
Source: BMC Evol Biol. 2015 Jun 30;15:127. doi: 10.1186/s12862-015-0398-x (PMC4487212; doi:10.1186/s12862-015-0398-x)
Supplement: Additional file 7: Table S1. — Primers used for cloning the 5′-flanking region of the microcephaly genes. [file 12862_2015_398_MOESM7_ESM.docx]

**Supplementary Table S1.** Primers used for cloning the 5’-flanking region of the microcephaly genes.

| Gene | Sequence |
| --- | --- |
| humanASPM prom_sense  humanASPM prom_antisense  chimpanzeeASPM prom_sense  chimpanzeeASPM prom_antisense  macaqueASPM prom_sense  macaqueASPM prom_antisense  marmosetASPM prom_sense  marmosetASPM prom_antisense  humanCDK5RAP2 prom_sense  humanCDK5RAP2 prom_antisense  chimpanzeeCDK5RAP2 prom_sense  chimpanzeeCDK5RAP2 prom_antisense  macaqueCDK5RAP2 prom_sense  macaqueCDK5RAP2 prom_antisense  marmosetCDK5RAP2 prom_sense  marmosetCDK5RAP2 prom_antisense  marmosetMCPH1 prom_sense  marmosetMCPH1 prom_antisense  humanWDR62 prom_sense  human WDR62 prom_antisense  chimpanzeeWDR62 prom_sense  chimpanzeeWDR62 prom_antisense  macaqueWDR62 prom_sense  macaqueWDR62 prom_antisense  marmosetWDR62 prom_sense  marmosetWDR62 prom_antisense | *5CCGCTCGAGCGAGAACTGGAAATGACTTGTGAGGCTCTG3*  *5CCCAAGCTTCATGGCAGATTCGAGACCCCTCC3*  5CCGCTCGAGGGAAATGACTTGTGAGGCTCTGCTC3  5CCCAAGCTTCATGGCAGATTCGAGACCCCTC3  5TCCCCCGGGACTGAAGTGACTTGTGAGGCTCTGTTC3  5CCCAAGCTTCATGGCAGCTTCGAGACTCCC3  5CCGCTCGAGCATAGAGCTTTACCTTGGGCCATGCTCTCT3  5CCCAAGCTTAGGACCGGAGGGGAAGTCGACTCCT3  5CCGCTCGAGATAATCGCGGGCTTTGGAAGC3  5CCCAAGCTTACTCTTTCAGAAAACTCCTTACCTGTTCTT3  5CCGCTCGAGTTGGAAGCCTAACTAAAGCTGAGAGAGAGAC3  5CCCAAGCTTCACACGCTCTACAAAGTACGTGGCG3  5CCGCTCGAGGGCAGAAAAAAAATACCACAGTGAG3  5CCCAAGCTTGAACACACAACACACAGTCCAAT3  5CCGCTCGAGCAGTGGCCCAGGCCTATAATCCCA3  5CCCAAGCTTGGCCACGTTAGAGCAAAACCCCATC3  5CGGGGTACCGGTGGCAGAAAGACAAATAGCTCAATG3  5CTAGCTAGCAGGGAGCAGGAAGTTCCTCACCTTT3  5CCGCTCGAGCAACATAGGGTGATCCCATCTCTCTC3  5CCCAAGCTTGCAGCTTCTCACCCGGTTCTG3  5CCGCTCGAGAGACTAGACTGGACAACATAGCGTGATCC3  5CCCAAGCTTAGTTCTCAGGACGGGACTAGGGGA3  5CCGCTCGAGGGTTCTTTGTGTCCAGCAGGGGC3  5CCCAAGCTTGGACTGGCCAGGGACTGATAGTCG3  5CGGGGTACCCGTGCCTTGGTCCCAGCTGCTT3  5CTAGCTAGCGAACTCCTGCCATGACAGAGGGCAG3 |

*Restriction enzyme sites located within the PCR primers are underlined. *Xho*Ⅰsite: CTCGAG; *Hind*Ⅲ site: AAGCTT. *Kpn*Ⅰsite:GGTACC; NheⅠ site:GCTAGC;
