# Supplementary material for: Estrogen regulation of microcephaly genes and evolution of brain sexual dimorphism in primates
Source: BMC Evol Biol. 2015 Jun 30;15:127. doi: 10.1186/s12862-015-0398-x (PMC4487212; doi:10.1186/s12862-015-0398-x)
Supplement: Additional file 8: Table S2. — Primers used for generating estrogen receptor binding site mutants of the MCPH gene promoter constructs. [file 12862_2015_398_MOESM8_ESM.docx]

**Supplementary Table S2.** Primers used for generating estrogen receptor binding site mutants of the MCPH gene promoter constructs.

| haspm-tgacc660F_sense  haspm-tgacc660R_antisense  haspm-ggtca1900F_sense  haspm-ggtca1900R_antisense  hcdk5rap2-tgacc1280F_sense  hcdk5rap2-tgacc1280R_antisense  hcdk5rap2-tgacc1360F_sense  hcdk5rap2-tgacc1360R_antisense  hcdk5rap2-tgacc1800F_sense  hcdk5rap2-tgacc1800R_antisense  hcdk5rap2-ggtca1480F_sense  hcdk5rap2-ggtca1480R_antisense  hcdk5rap2-ggtca1510F_sense  hcdk5rap2-ggtca1510R_antisense  hcdk5rap2-ggtca930F_sense  hcdk5rap2-ggtca930R_antisense  hmcph1-tgacc1000F_sense  hmcph1-tgacc1000R_antisense  hmcph1-tgacc1600F_sense  hmcph1-tgacc1600R _antisense  hmcph1-ggtca1760F_sense  hmcph1-ggtca1760R_antisense  hwdr62-tgacc310F_sense  hwdr62-tgacc310R_antisense  hwdr62-ggtca1280F_sense  hwdr62-ggtca1280R_antisense | 5CCTACTTAGCTGTATGTATTCCTCATCTGTAAGATA3  5TATCTTACAGATGAGGAATACATACAGCTAAGTAGG3  5CCCCTGAGGGGAAGCGCGTAAGTCCCGGAT3  5ATCCGGGACTTACGCGCTTCCCCTCAGGGG3  5TTGCTGGCCCTCAGTTTTCACAGGTGCCTTTGAGTCC3  5GGACTCAAAGGCACCTGTGAAAACTGAGGGCCAGCAA3  5GACTGGAGTGGGAGTGCTGTACTGGCTCATGGGAATGAGT3  5ACTCATTCCCATGAGCCAGTACAGCACTCCCACTCCAGTC3  5CTGCGTGCGCCTCCGCCGCCTCGTTTCCGT3  5ACGGAAACGAGGCGGCGGAGGCGCACGCAG3  5TATCTGAGTTTCAACTTAAAATCCCAAAGTTTTGGGGTC3  5GACCCCAAAACTTTGGGATTTTAAGTTGAAACTCAGATA3  5GTCAAATCCCAAAGTTTTGGTTATTCTAGAAAAGCAAACA3  5TGTTTGCTTTTCTAGAATAACCAAAACTTTGGGATTTGAC3  5GGTGGGCAGATCACGAGGAGATTGAGACCATC3  5GATGGTCTCAATCTCCTCGTGATCTGCCCACC3  5CTAAACTTTTTTTACCACCTAAAAGTAATTGGTTTATATA3  5TATATAAACCAATTACTTTTAGGTGGTAAAAAAAGTTTAG3  5GGTTCACCCCGCGGTGAGTCCTGGGATG3  5CATCCCAGGACTCACCGCGGGGTGAACC3  5GGTCTGAGATCTCGGCCCTGTGGGGGTCTG3  5CAGACCCCCACAGGGCCGAGATCTCAGACC3  5AGGCTGGTCTCGAACTCCTCAGGTGATCTGCCCGCC3  5GGCGGGCAGATCACCTGAGGAGTTCGAGACCAGCCT3  5CGCTGAGTTTTGCCGGCGGCTGCACTTTGG3  5CCAAAGTGCAGCCGCCGGCAAAACTCAGCG3 |
| --- | --- |
