# Supplementary material for: Estrogen regulation of microcephaly genes and evolution of brain sexual dimorphism in primates
Source: BMC Evol Biol. 2015 Jun 30;15:127. doi: 10.1186/s12862-015-0398-x (PMC4487212; doi:10.1186/s12862-015-0398-x)
Supplement: Additional file 9: Table S3. — Primers used for real-time quantitative PCR. [file 12862_2015_398_MOESM9_ESM.docx]

**Supplementary Table S3.** Primers used for real-time quantitative PCR.

| Gene | Sequence |
| --- | --- |
| humanMCPH1 _sense  humanMCPH1 _antisense  humanGAPDH_sense  humanGAPDH_antisense  humanASPM_sense  humanASPM_antisense  humanWDR62_sense  humanWDR62_antisense  humanCDK5RAP2_sense  humanCDK5RAP2_antisense | 5’-AGCGCAATGGAGAAGAGATTACAAG-3’  5’-ATCCTGAGTTTCCACAAAGATCATC-3’  5’-ACCACAGTCCATGCCATCAC-3’  5’-TCCACCACCCTGTTGCTGTA-3’  5TGCAGTGGGTGAACATGAAAA3  5CGAAGAGGGTGTTACCTCGTTT3  5GCGAGCTGCACAACAACATC3  5GAGGCCCGAGTAGGACACA3  5AAGATGCTCGAAAGAAGGTGC3  5TTTCCAAACGCAACCGAAGAG3 |
