# Supplementary material for: Estrogen regulation of microcephaly genes and evolution of brain sexual dimorphism in primates
Source: BMC Evol Biol. 2015 Jun 30;15:127. doi: 10.1186/s12862-015-0398-x (PMC4487212; doi:10.1186/s12862-015-0398-x)
Supplement: Additional file 10: Table S4. — The brain samples of different developmental stages used in this study from published data at www.brainspan.org. [file 12862_2015_398_MOESM10_ESM.docx]

**Supplementary Table S4.**  The brain samples of different developmental stages used in this study from published data at [www.brainspan.org](http://www.brainspan.org).

| **Development Stage** | **Male Number** | **Female Number** |
| --- | --- | --- |
| 13pcw | 28 | 16 |
| 16pcw | 40 | - |
| 19pcw | - | 11 |
| 21pcw | 15 | 16 |
| 24pcw | 16 | - |
| 25pcw | - | 2 |
| 26pcw | - | 5 |
| 1yrs | - | 16 |
| 2yrs | - | 16 |
| 3yrs | 14 | 13 |
| 4yrs | 16 | - |
| 8yrs | - | 28 |
| 11yrs | - | 14 |
| 13yrs | - | 16 |
| 15yrs | 15 | - |
| 18yrs | 13 | - |
| 19yrs | - | 16 |
| 21yrs | - | 16 |
| 23yrs | 16 | - |
| 30yrs | - | 16 |
| 36yrs | 16 | - |
| 37yrs | 15 | - |
| 40yrs | 16 | 15 |
